# Selenium Deficiency and Chronic Pancreatitis: Disease Mechanism and Potential for Therapy

**DOI:** 10.1155/1999/97140

**Published:** 1999-07

**Authors:** David J. Bowrey, Gareth J. Morris-Stiff, Malcolm C. A. Puntis

**Affiliations:** University Department of Surgery University Hospital of Wales Heath Park Cardiff CF4 4XN United Kingdom

## Abstract

*Background:*  It has been suggested that antioxidant deficiency may play a role in the pathogenesis of chronic pancreatitis. The aim of this review was to analyse the evidence for this relationship and to consider the role of antioxidant supplementation in the treatment of chronic pancreatitis.

*Methods:* Medline review of all English language publications for the years 1966–1998.

*Results and Conclusions:* There is evidence that patients with chronic pancreatitis have enhanced levels of free radical production, cytochrome P450 induction and antioxidant deficiencies, in particular selenium. The limited published literature in this field suggests that dietary antioxidant supplementation may ameliorate the pain associated with chronic pancreatitis, diminish the frequency of acute exacerbations and reduce the requirement for pancreatic surgery. These findings await confirmation by a large prospective placebo-controlled study.


*HPB Surgery*, 1999, Vol. 11, pp. 207–216
Reprints available directly from the publisher
Photocopying permitted by license only

© 1999 OPA (Overseas Publishers Association) N.V.
Published by license under
the Harwood Academic Publishers imprint,
part of The Gordon and Breach Publishing Group.
Printed in Malaysia.

### Selenium Deficiency and Chronic Pancreatitis: Disease Mechanism and Potential for Therapy

DAVID J. BOWREY*, GARETH J. MORRIS-STIFF† and MALCOLM C. A. PUNTIS

*University Department of Surgery, University Hospital of Wales, Heath Park, Cardiff, CF4 4XN, United Kingdom*

*(Received 13 August 1998; In final form 1 November 1998)*

***Background:*** **It has been suggested that antioxidant deficiency may play a role in the pathogenesis of chronic pancreatitis. The aim of this review was to analyse the evidence for this relationship and to consider the role of antioxidant supplementation in the treatment of chronic pancreatitis.**

***Methods:*** **Medline review of all English language publications for the years 1966–1998.**

***Results and Conclusions:*** **There is evidence that patients with chronic pancreatitis have enhanced levels of free radical production, cytochrome P450 induction and antioxidant deficiencies, in particular selenium. The limited published literature in this field suggests that dietary antioxidant supplementation may ameliorate the pain associated with chronic pancreatitis, diminish the frequency of acute exacerbations and reduce the requirement for pancreatic surgery. These findings await confirmation by a large prospective placebo-controlled study.**

*Keywords:* Chronic pancreatitis, selenium, antioxidant, deficiency, replacement therapy

### INTRODUCTION

Selenium was first identified in 1817 by the Swedish scientist Jöns Jacob Berzelius as a red deposit on the walls of a lead chamber used in the production of sulphuric acid [1]. It derives its name from the Greek moon goddess Selene and in its chemical properties, it shares many similarities with sulphur [1]. It is a ubiquitous element and is widely distributed in the earth's crust, in the environment and the food chain. It occurs naturally in an inorganic state as selenite and selenate, and in an organic, state as selenoamino acids [1,2]. The latter are present particularly in plants and constitute the principal source of dietary selenium [3]. The selenium content of plants is directly related to the amount of the element present within the soil in which the plants grow. This in turn is inversely related to the annual rainfall of the locality, as selenium is leached out of soil by virtue of its aqueous solubility [4]. There is a wide geographic variation in the amount of selenium in soil, with low levels found in Denmark, Finland, New Zealand, Australia, Siberia, Korea and certain parts of north-eastern and south central China. Regions with a high soil selenium content include parts

*Tel.: 0044-1222-746536, Fax: 0044-1222-761623, e-mail: bowrey@cf.ac.uk
†Corresponding author.

of Western China and several western states of the United States of America [1, 4].

This review examines the role of selenium in the pathogenesis of chronic pancreatitis and its potential role in the treatment of this condition. It is based upon literature obtained from a Medline search covering the period 1966 to 1998 which identified all English language publications using the keywords selenium, antioxidant deficiency and chronic pancreatitis.

### SELENIUM PHYSIOLOGY

In food, selenium is usually present in the form of organic compounds such as selenomethionine, selenocysteine and selenotaurine. Foods with the highest selenium content are brazil nuts (254 μg/100 g) and crab meat (84 μg/100 g). The levels in vegetables and dairy products are much lower (<2 μg/100 g) [5–7]. Selenium, in the form of selenite, selenate, selenocysteine and selenomethionine is absorbed in the duodenum. There is considerable variation in the absorption of selenium compounds from around 55% for selenite to 90% for selenomethionine. Absorption is inhibited by methionine, heavy metals (cadmium, arsenic, mercury) and food rich in fibre, while it is promoted by vitamins A, C and E [5]. In blood, absorbed selenium is rapidly taken up by red blood cells where it is reduced by thiols to hydrogen selenide, released into the plasma and transported bound to alpha and beta globulins [5]. Selenium occurs in all human tissue, in particular the liver (0.63 μg/g) and kidney (0.39 μg/g) [2].

### DIETARY SELENIUM

Based upon a series of depletion–repletion studies carried out in Chinese males the United States set the recommended daily intakes for selenium at 70 μg/day for men and 55 μg/day for women [8, 9]. Recommended daily intakes for other groups were extrapolated from the adult range on the basis of body weight, with a factor added for growth. The United Kingdom Department of Health has recommended daily selenium intakes of 1.0 μg/kg based upon the depletion–repletion studies and the amount of selenium required to maintain glutathione peroxidase at its plateau of activity [10].

Based upon dietary estimates Thorn *et al.* [7] found in 1978 that the intake of selenium by the United Kingdom population was adequate. A more recent study by the Ministry of Agriculture, Fisheries and Food [6, 11] has reported an average intake of only 34 μg/day, approximately 50% of the recommended daily intake. This decline in dietary selenium intake has resulted from a reduction in imports of selenium-rich wheat from North America following the United Kingdom's entry into the European Union and also as a result of changes in breadmaking technology.

### SELENOPROTEINS

The biochemical function of selenium was unknown until 1973, when it was discovered that it was an integral component of the active site of the mammalian enzyme glutathione peroxidase [12]. It is now well established that selenium is a highly specific component of enzymes in both eukaryotes and prokaryotes. The selenium-dependent enzymes are redox catalysts and most contain selenium in the form of selenocysteine residues [4]. Eukaryotic selenoenzymes identified include the glutathione peroxidases, tetraiodothyronine 5-deiodinase, selenoprotein P, sperm capsule selenoprotein and selenoprotein W [4, 13, 14].

The most important mammalian selenoproteins are the glutathione peroxidases. These enzymes, together with superoxide dismutase and catalase form part of the cellular defence mechanism against reactive molecules and free radicals which cause lipid peroxidation and interact with intra-cellular macromolecules [4, 13–15]. The toxic oxygen derivatives, which are neutralised by the antioxidants are produced by normal cellular metabolic activity and by a variety of injurious agents, including drugs, toxins, and irradiation. In conjunction with vitamins A, C and E, glutathione peroxidase functions as a cellular protector against oxidative damage, by catalysing the breakdown of hydrogen peroxide and fatty acyl lipid peroxides, in the presence of reduced glutathione, to water and the corresponding alcohols. Such peroxides are a source of potentially damaging free radicals which can cause peroxidation of polyunsaturated fatty acids in the cell membrane [4, 13, 14].

It is postulated that deficiencies of micronutrients such as selenium reduce the ability of these antioxidant free radical scavenging enzymes leading to a situation referred to as oxidative stress [16].

### ENDEMIC SELENIUM DEFICIENCY AND HUMAN DISEASE

The evidence for the effects of selenium deficiency in humans come from indirect evidence from China and from findings in patients receiving total parenteral nutrition [1]. Two diseases found in areas of low-selenium content soil in China have been ascribed to selenium defeciency. Keshan disease is a cardiomyopathy seen commonly in childhood characterised by multifocal necrosis and fibrous replacement of the myocardium. Several large-scale studies have shown that prophylactic treatment of the population with oral supplements have resulted in virtual eradication of the condition in endemic areas. Kashin-Beck disease (osteoarthritis deformans endemica) is another selenium-responsive endemic disease occurring in Northern China, North Korea, and eastern Siberia. The disease affects principally children and is characterised by a chronic disabling degenerative osteoarthritis [1]. It is interesting that there are no reports so far of a high prevalence of chronic pancreatitis in these Chinese patients. However, in the Indian Subcontinent there are increasing reports of a high prevalence of chronic pancreatitis [17, 18]. Evidence of clinically significant selenium deficiency from the developed world is confined to case reports of patients receiving parenteral nutrition, in whom symptoms resolved upon selenium supplementation. The syndromes reported included myalgia and myositis; and cardiomyopathy. Following these early observations selenium supplementation of parenteral nutrition has become routine practice [1].

### CHRONIC PANCREATITIS

Chronic pancreatitis is defined as a persistent, usually progressive, inflammatory condition characterised by irregular sclerosis of the gland with destruction and loss of exocrine parenchyma [19]. It is less frequent than acute pancreatitis and shows considerable geographical variation in its incidence. In the United Kingdom the annual incidence is in the order of 3 per 100,000 population, with 60–85% being alcohol-related [19]. The condition affects males more frequently and has its onset at a median age of 40 years. The condition is seen more commonly in tropical countries where protein malnutrition in infancy is endemic. In these countries the disease affects both genders with equal frequency and occurs in patients below the age of 20 years [19].

### OXIDATIVE STRESS

Oxidative stress refers to a situation that is characterised by a relative deficiency in antioxidant levels compared to the rate of free radical production and thus it is a state of potential tissue injury. The evidence that chronic pancreatitis is related to oxidative stress is indirect as free radicals are undetectable *in-vivo* due to their short half-life. There is a substantial body of evidence showing antioxidant deficiency and increased levels of free radical oxidation products in patients with chronic pancreatitis.

### SELENIUM DEFICIENCY IN CHRONIC PANCREATITIS

Initial evidence suggesting deficiencies of antioxidants, in particular selenium, in chronic pancreatitis was derived from the dietary analysis of index patients. Rose *et al.* [20] compared the dietary histories of 15 patients with idiopathic chronic pancreatitis and found that index patients had significantly reduced ingestion of selenium; vitamins C and E; and riboflavin, than did controls. Further, theophylline clearance testing, employed as an index of cytochrome P450 activity and by inference antioxidant demand detected significant differences between patients with chronic pancreatitis and controls. There was an inverse relationship between theophylline clearance and selenium intake, pancreatitics having a more rapid drug clearance and lower selenium intake.

Subsequently, antioxidant deficiencies, in particular selenium were demonstrated by *in-vivo* estimation of serum selenium from index patients. Mathew *et al.* [21] studied four groups of patients: adults with hereditary pancreatitis ($n = 14$), relatives of the adults with hereditary pancreatitis ($n = 11$), children with chronic pancreatitis ($n = 7$) and a control population ($n = 65$). Controls had significantly higher serum selenium and glutathione peroxidase levels compared to each of the three other groups. Adults with hereditary pancreatitis had significantly reduced serum selenium levels compared to their relatives (median 11.7 *vs.* 14.1 μg/dl), but significantly greater selenium levels compared to children with chronic pancreatitis (median 11.7 *vs.* 9.33 μg/dl). These observations suggest that patients with hereditary pancreatitis may have dietary deficiencies of antioxidants that may compound an inherited predisposition to this condition. Interestingly, the paediatric patients had the lowest selenium levels of any group suggesting that the more severe the antioxidant deficiency, the earlier the age at which the condition becomes manifest.

Van Gossum *et al.* [22] found significantly reduced levels of the following antioxidants: selenium (median 54 *vs.* 87 μg/l), vitamin A (median 30 *vs.* 49 μg/dl) and vitamin E (8 *vs.* 16 mg/l) in patients with alcohol-related chronic pancreatitis ($n = 35$) compared to healthy controls ($n = 14$). Sub-group analysis revealed that vitamin E levels were significantly lower in patients with steatorrhoea compared to patients in whom this feature was not observed. No relationship between pancreatic exocrine function and selenium deficiency was seen, implying that selenium deficiency was primary to the pathophysiology of the condition, and not secondary to pancreatic insufficiency.

Braganza *et al.* [23] studied 37 patients with chronic pancreatitis (23 idiopathic, 14 alcohol-related) and compared serum selenium levels to 41 controls. The mean selenium levels were 93 μg/l (range 48 – 156) for the patient population and 117 μg/l (range 81 – 161) for the control population ($p < 0.001$). For pancreatitics no difference was observed for selenium levels between patients with alcohol-related pancreatitis and patients with idiopathic pancreatitis. However, when patients were classified according to the presence or absence of pancreatic pain, the subgroup of patients with pancreatic pain had significantly lower selenium levels (mean 74 μg/l) compared to patients in remission from pain (mean level 107 μg/l) and patients who had never experienced pain (mean level 102 μg/l).

### Tropical Pancreatitis

Several studies [17, 18, 24] have compared the antioxidant profiles from chronic pancreatitics in the United Kingdom (temperate-zone pancreatitis) and patients with chronic pancreatitics from India and South Africa (tropical pancreatitis) in an attempt to identify differences that might explain the different demographics of the condition in the two settings [19].

Explanations proposed to account for the differences between sporadic chronic pancreatitis in the developed world and the endemic calcific chronic pancreatitis occurring in the tropics have centred upon the extent of antioxidant deficiencies. In a comparison of healthy controls from Manchester, UK and Madras, India, Braganza *et al.* [18] found that healthy volunteers from India had significantly lower levels of beta-carotene (medians 40 *vs.* 98 μg/l) and ascorbic acid (medians 1.6 *vs.* 13 mg/l) compared with the British control population. Levels of selenium (medians 117 *vs.* 119 μg/l), alpha-tocopherol (medians 4.8 *vs.* 4.7 mmol/mol.cholesterol) and vitamin C (medians 12 *vs.* 13 mg/l) were similar in the two control groups for both locations. At both sites patients with chronic pancreatitis had significantly lower levels of selenium, alpha-tocopherol and vitamin C compared to controls derived from the local population. Pancreatitics from Manchester also showed reduced levels of beta-carotene and ascorbic acid compared to local controls.

Yadav *et al.* [17] studied controls and chronic pancreatitis patients from Madras, India and Manchester, United Kingdom and found that for both locations chronic pancreatitics had reduced serum levels of selenium compared to a control population. Further, there was no difference in the absolute selenium levels between equivalent populations in the two locations despite a significantly higher prevalence of diabetes in the Indian patients. In a similar study comparing the Manchester controls with controls from Soweto, South Africa, Segal *et al.* [24] found significantly reduced levels of vitamin C (medians 3 *vs.* 13 mg/l) and selenium (medians 105 *vs.* 119 μg/l) in the South African group. Levels of beta-carotene and alpha-tocopherol were comparable in the two populations.

It has been suggested that the differences in the antioxidant profiles for controls and chronic pancreatitics from Western and tropical populations are related to variations in culinary practices at these locations. In South Africa this is considered to be due to a poor dietary intake of citrus fruit and in India, the degradation of vitamin C and beta-carotene following prolonged frying of vegetables at high temperatures may be responsible.

### ENHANCED FREE RADICAL PRODUCTION IN CHRONIC PANCREATITIS

The evidence that free radical production is increased in patients with chronic pancreatitis is derived from the observations that pancreatitics have induction of the hepatic and pancreatic mono-oxygenases and that their bile contains high levels of free radical oxidation products.

The cytochrome P450 system of mono-oxygenases are intimately involved in the metabolism of lipophilic substrates such as hydrocarbons [25]. The enzymes generate oxygen-free radicals from molecular oxygen and use them to metabolise their substrates by controlled release of superoxide, and its dismutation product hydrogen peroxide. The hydrogen peroxide in turn initiates a low grade peroxidation of membrane lipids which appears to be an essential physiological process. Upon exposure to exogenous agents, the activities of cytochromes P450 increase as part of a protective mechanism. This defence mechanism can be detrimental if antioxidant stores and depleted, and if agents that undergo P450 metabolism (*e.g.*, hydrocarbons) are given concomitantly. This is exemplified by animal experiments showing that following the induction of the cytochromes P450 by alcohol, the hepatotoxicity of hydrocarbons is accentuated [26]. Further, a case-control study [27] of patients with chronic pancreatitis has shown that they have increased exposure to hydrocarbons, in particular diesel exhaust fumes compared to controls. These environmental chemicals would act in a manner similar to alcohol and induce cytochromes P450.

Several studies have shown that the cytochrome P450 enzyme system is induced in patients suffering from chronic pancreatitis [28–31]. Foster *et al.* [28] employed immunohistochemistry using antibodies raised against four phase I enzymes (metabolism) and one phase II enzyme (conjugation) and compared the staining patterns in pancreatic and hepatic biopsies from patients with chronic pancreatitis ($n = 6$) and pancreatic cancer ($n = 10$) with a control population of organ donors ($n = 7$). Both hepatic and pancreatic biopsy specimens from patients with chronic pancreatitis and pancreatic carcinoma showed enhanced staining for cytochrome P450 compared to the staining pattern observed in the control biopsies. Of note was the observation of cytochrome P450 induction in the islets of Langerhans. The authors commented that this may indicate a role in the development of type II diabetes mellitus in this group of patients.

Inference of cytochrome P450 enzyme family induction is derived from theophylline clearance studies in patients with chronic pancreatitis. Acheson *et al.* [29] studied 71 patients with chronic pancreatitis and found that the theophylline clearance was significantly higher in patients with idiopathic and alcohol-related chronic pancreatitis compared to controls. Uden *et al.* [30] compared patients with chronic pancreatitis to a group of epileptic patients taking long-term anticonvulsants, known inducers of the cytochrome P450 enzyme system and found that pancreatitics had a similar level of enzyme induction to anticonvulsant-treated patients.

Comparison of the composition of secretin-simulated bile from patients with pancreatic disease (chronic pancreatitis [$n = 11$], pancreatic cancer [$n = 5$], recurrent acute pancreatitis [$n = 1$]) to controls revealed that patients with pancreatic disease had higher levels of free radical oxidation products [32]. Guyan *et al.* [33] compared the levels of free radical oxidation products in 25 patients with pancreatitis (15 chronic, 10 acute) and 25 controls. Patients with chronic pancreatitis had significantly greater bile concentration of 9 *cis*, 11 *trans*-linoleic acid, conjugated dienes and ultraviolet fluorescence products compared to controls. Patients with acute pancreatitis had significantly elevated excretions of 9 *cis*, 11 *trans*-linoleic acid and conjugated dienes but not ultraviolet fluorescence products compared to controls. These observations suggest that the degree of oxidative stress is greatest in patients with chronic pancreatitis and intermediate in patients with acute pancreatitis.

The phospholipid component of secretin-stimulated bile is derived almost exclusively from hepatic bile and not pancreatic juice [34]. The occurrence of high levels of free radicals activity in the bile of patients with pancreatitis would lend support to the hypothesis that biliary reflux into the pancreatic duct is the cause of pancreatitis. However, the failure of biliary diversion to prevent episodes of pancreatitis contradicts this theory [31]. Sandilands *et al.* [31] obtained liver and pancreatic biopsies per-operatively from 4 patients with chronic pancreatitis managed by biliary diversion and found that both tissues demonstrated similar histological findings. The liver biopsies showed accumulation of microvesicular fat and lipofuscin and the pancreatic biopsies revealed accumulation of microvesicular fat. The pancreas and liver, with a common embryological origin from the endoderm of the duodenum [35] react in a similar fashion to oxidative stress. The liver, with its greater conjugation enzyme potential is less susceptible to free radical injury than the pancreas.

### SELENIUM REPLACEMENT THERAPY IN CHRONIC PANCREATITIS

The first evidence suggesting a beneficial role for antioxidant supplementation in pancreatitis was from Braganza *et al.*, in 1987 [36]. The patient, a 61 year old female who suffered from recurrent acute pancreatitis over a period of two years experienced no further episodes at a follow-up time of two years following treatment with selenium (200 μg), vitamins A, C and E. Subsequent case reports from the same unit afforded further evidence about the therapeutic value of antioxidants in the treatment of chronic calcific pancretatitis in a 10 year old boy and idiopathic chronic pancreatitis in 4 adult male patients [31, 37]. This work served as pilot studies for a randomised controlled trial by Dr. Braganza's group. In this study [38], the value of antioxidant therapy was assessed in 20 patients (8 idiopathic chronic pancreatitis, 7 alcohol-related chronic pancreatitis, 5 recurrent acute pancreatitis) entered into a 20 week double-blind double-dummy cross-over trial. Antioxidant supplementation comprised 600 μg organic selenium, 9000 iu beta-carotene, 0.54 g vitamin C, 270 iu vitamin E and 2 g methionine. Six patients experienced an attack of pancreatitis whilst on placebo, whilst none receiving active treatment did. Further, pain scores assessed using visual analogue scores were significantly improved whilst patients received active treatment compared to the baseline pain scores.

In an attempt to identify the active ingredient in the global antioxidant therapy described in the above study, Bilton *et al.* [39] studied the effects of the active component of methionine, S-adenosyl-methionine in 20 patients with chronic pancreatitis in a placebo-controlled trial performed over a period of 20 weeks. As this treatment alone was ineffective, the investigators studied the effect of co-administering selenium and beta-carotene, with the S-adenosyl-methionine. This treatment also proved ineffective and the authors concluded that the global antioxidant therapy was required to achieve a beneficial therapeutic response in patients with chronic pancreatitis. This should include S-adenosyl methionine, selenium and vitamins A, C and E.

There is only anecdotal evidence [40] of the potential reduction in morbidity and the financial savings of administering global antioxidant therapy to all patients with chronic pancreatitis. An audit at Manchester of 103 patients treated with antioxidant therapy revealed that at a follow-up of up to 9 years, 75 patients were pain-free and that 27 patients had substantial reductions in their pain. Only 7 patients required surgery, pseudocyst drainage in 6 patients and cholecystectomy in one patient.

### CONCLUSIONS

It is likely that there is a common aetiology to chronic pancreatitis related to abnormal processing of oxygen free radical products generated by a range of xenobiotics however, it is not known how alcohol is related to this. There is little doubt that patients with chronic pancreatitis have low levels of selenium compared with non-pancreatitics however it is uncertain at present whether this is the causal or merely an effect of the disease process. It may be that oxidative stress is triggered, in an individual of antioxidant deficient status, by ischaemic damage secondary to dilatation of the intrapancreatic ductal system [41]. It has recently been demonstrated in a feline model that an obstructed ductal system within a fibrous pancreas causes a "compartment syndrome". This leads to a reduced pancreatic blood flow and ischaemiac damage, which is turn may generate further free radical species.

As China opens its doors to the West, the results of large-scale population studies in endemic regions will no doubt clarify the relationship between selenium deficiency and chronic pancreatitis.

### *References*

[1] Lockitch, G. (1989). Selenium: clinical significance and analytical concepts. *Critical Reviews in Clinical Laboratory Sciences*, **6**, 483–541.

[2] Dickson, R. C. and Tomlinson, R. H. (1967). Selenium in blood and human tissues. *Clinical Chimica Acta*, **16**, 311–321.

[3] Bronzetti, G. and della Croce, C. (1993). Selenium: its important roles in life and contrasting aspects. *Journal of Environmental Pathology, Toxicology and Oncology*, **12**, 59–71.
[4] Stadtman, T. C. (1994). Selenium biochemistry-selected topics. *Advances in Inorganic Biochemistry*, **10**, 157–175.
[5] Buss, D. H. and Rose, H. J. (1992). Dietary intake of nutrient trace elements. *Food Chemistry*, **43**, 209.
[6] Ministry of Agriculture, Fisheries and Farming (1994). The British diet: finding the facts 1989–1993. *40th Report of the Steering Group on Chemical Aspects of Food Surveillance*, HMSO, London.
[7] Thorn, J., Robertson, J., Buss, D. H. and Bunton, N. G. (1978). Trace nutrients. Selenium in British food. *British Journal of Nutrition*, **39**, 391–396.
[8] Yang, G., Ge, K., Chen, J. and Chen, X. (1988). Selenium related endemic diseases and the daily requirement of humans. *World Reviews of Nutrition and Dietetics*, **55**, 98–152.
[9] Yang, G., Yin, R., Zhou, L., Gu, L., Yan, B. and Lui, Y. (1989). Studies of safe and maximal daily dietary Se-intake in a seleniferous area of China. Part II: Relations between Se-intake and the manifestation of clinical signs and certain biochemical alterations in blood and urine. *Journal of Trace Elements and Electrolytes in Health and Disease*, **3**, 123–130.
[10] Department of Health (1991). Dietary reference values for food energy and nutrients of the United Kingdom: report on the panel on dietary reference numbers of the committee of medical aspects of food policy. *Report on Health and Social Subjects*, London, HMSO, p. 41.
[11] Barclay, M. N. I., MacPherson, A. and Dixon, J. (1995). Selenium content of a range of UK foods. *Journal of Food Composition and Analysis*, **8**, 307–318.
[12] Rotruck, J. T., Pope, A. L., Ganther, H. E., Swanson, A. B., Hafeman, D. G. and Hoekstra, W. G. (1973). Selenium: biochemical role as a component of glutathione peroxidase. *Science*, **179**, 588–590.
[13] Zachara, B. A. (1992). Mammalian selenoproteins. *Journal of Trace Elements and Electrolytes in Health and Disease*, **6**, 137–151.
[14] Arthur, J. R. and Beckett, G. J. (1994). New aspects of micronutrients in at risk groups. New metabolic roles for selenium. *Proceedings of the Nutrition Society*, **53**, 615–624.
[15] Gutteridge, J. M. C. and Halliwell, B. (1990). The measurement and mechanism of lipid peroxidation in biological systems. *Trends in Biochemical Science*, **15**, 129–135.
[16] Braganza, J. M. (1996). The pathogenesis of chronic pancreatitis. *Quarterly Journal of Medicine*, **89**, 243–250.
[17] Yadav, S., Day, J. P., Mohan, V., Snehalatha, C. and Braganza, J. M. (1991). Selenium and diabetes in the tropics. *Pancreas*, **6**, 528–533.
[18] Braganza, J. M., Schofield, D., Snehalatha, C. and Mohan, V. (1993). Micronutrient antioxidant status in tropical compared with temperate-zone chronic pancreatitis. *Scandinavian Journal of Gastroenterology*, **28**, 1098–1104.
[19] Rode, J. (1992). The exocrine pancreas. In: McGee, J. O., Isaacson, P. G. and Wright, N. A., *Oxford Textbook of Pathology*, Oxford University Press, Oxford, pp. 1429–1448.
[20] Rose, P., Fraine, E., Hunt, L. P., Acheson, D. W. K. and Braganza, J. M. (1986). Dietary antioxidants and chronic pancreatitis. *Human Nutrition: Clinical Nutrition*, **40C**, 151–164.
[21] Mathew, P., Wyllie, R., Van Lente, F., Steffen, R. M. and Kay, M. H. (1996). Antioxidants in hereditary pancreatitis. *American Journal of Gastroenterology*, **91**, 1558–1562.
[22] Van Gossum, A., Closset, P., Noel, E., Cremer, M. and Neve, J. (1996). Deficiency in antioxidant factors in patients with alcohol-related chronic pancreatitis. *Digestive Diseases and Sciences*, **41**, 1225–1231.
[23] Braganza, J. M., Hewitt, C. D. and Day, J. P. (1993). Serum selenium in patients with chronic pancreatitis: lowest values during painful exacerbations. *Pancreas*, **2**, 80–85.
[24] Segal, I., Gut, A., Schofield, D., Shiel, N. and Braganza, J. M. (1995). Micronutrient antioxidant status in black South Africans with chronic pancreatitis: opportunity for prophylaxis. *Clinica Chimica Acta*, **239**, 71–79.
[25] Koop, D. R. (1992). Oxidative and reductive metabolism by cytochrome P450 2E1. *FASEB Journal*, **6**, 724–730.
[26] Lindros, K. O., Cai, Y. A. and Penttila, K. E. (1990). Role of ethanol-inducible cytochrome P450 IIE1 in carbon tetrachloride-induced damage to centrilobular hepatocytes from ethanol treated rats. *Hepatology*, **12**, 1092–1097.
[27] McNamee, R., Braganza, J. M., Hogg, J., Leck, I., Rose, P. and Cherry, N. M. (1994). Occupational exposure to hydrocarbons and chronic pancreatitis: a case-referent study. *Occupational and Environmental Medicine*, **51**, 631–637.
[28] Foster, J. R., Idle, J. R., Hardwick, J. P., Bars, R., Scott, P. and Braganza, J. M. (1993). Induction of drug-metabolizing enzymes in human pancreatic cancer and chronic pancreatitis. *Journal of Pathology*, **169**, 457–463.
[29] Acheson, D. W. K., Hunt, L. P., Rose, P., Houston, J. B. and Braganza, J. M. (1989). Factors contributing to the accelerated clearance of theophylline and antipyrine in adults with exocrine pancreatic disease. *Clinical Science*, **76**, 377–385.
[30] Uden, S., Acheson, D. W. K., Reeves, J., Worthington, H. V., Hunt, L. P., Brown, S. and Braganza, J. M. (1988). Antioxidants, enzyme induction, and chronic pancreatitis: a reappraisal following studies in patients on anticonvulsants. *European Journal of Clinical Nutrition*, **42**, 561–569.
[31] Sandilands, D., Jeffrey, I. J. M., Haboubi, N. Y., MacLennan, I. A. M. and Braganza, J. M. (1990). Abnormal drug metabolism in chronic pancreatitis: treatment with antioxidants. *Gastroenterology*, **98**, 766–772.
[32] Braganza, J. M., Wickens, D. L., Cawood, P. and Dormandy, T. L. (1983). Lipid peroxidation (free-radical-oxidation) products in bile from patients with pancreatic disease. *Lancet*, **2**, 375–378.
[33] Guyan, P. M., Uden, S. and Braganza, J. M. (1990). Heightened free radical activity in pancreatitis. *Free Radical Biology and Medicine*, **8**, 347–354.
[34] Taj, M., Rose, P., Hunt, L. P., Smith, G. N. and Braganza, J. M. (1986). Hypersecretion of biliary fatty acids in patients with exocrine pancreatic disease. *International Journal of Pancreatology*, **1**, 309–326.
[35] Yeo, C. L. and Cameron, J. L. (1991). The pancreas. In: Sabiston, D. C. (Ed.) *Textbook of Surgery. The Biological Basis of Modern Surgical Practice*. W. B. Saunders Company, Philadelphia, pp. 1076–1107.

[36] Braganza, J. M., Jeffrey, I. J., Foster, J. and McCloy, R. F. (1987). Recalcitrant pancreatitis: eventual control by antioxidants. *Pancreas*, **2**, 489–494.
[37] Braganza, J. M., Thomas, A. and Robinso, A. (1988). Antioxidants to treat chronic pancreatitis in childhood? Case report and possible implications for pathogenesis. *International Journal of Pancreatology*, **3**, 209–216.
[38] Uden, S., Schofield, D., Miller, P. F., Day, J. P., Bottiglier, T. and Braganza, J. M. (1992). Antioxidant therapy for recurrent pancreatitis: biochemical profiles in a placebocontrolled trial. *Alimentary Pharmacology and Therapeutics*, **6**, 229–240.
[39] Bilton, D., Schofield, D., Mei, G., Kay, P. M., Bottiglieri, T. and Braganza, J. M. (1994). Placebo controlled trials of antioxidant therapy including S-adenosylmethionine in patients with recurrent nongallstone pancreatitis. *Drug Investigations*, **8**, 10–20.
[40] Whiteley, G. S. W., Kienle, A. P. B., McCloy, R. F. and Braganza, J. M. (1993). Long-term pain relief without surgery in chronic pancreatitis: value of antioxidant therapy. *Gastroenterology*, **104** (suppl), A343.
[41] Karankia, N. D., Widdison, A. L., Leung, F., Alvarez, C., Lutrin, F. J. and Reber, H. A. (1994). Compartment syndrome in experimental chronic obstructive pancreatitis: effect of decompressing the main pancreatic duct. *British Journal of Surgery*, **81**, 259–264.

### COMMENT ON "SELENIUM DEFICIENCY AND CHRONIC PANCREATITIS: DISEASE MECHANISM AN POTENTIAL FOR THERAPY"

Although much has been done to improve our understanding of the pathophysiology of chronic pancreatitis, there is still no therapy that counteracts the inflammatory process of the disease. One reason for this lack of knowledge is that one may assume that the etiology of chronic pancreatis multifactorial; selenium deficiency being one of these factors.

Most patients presenting with chronic pancreatitis in Europe, United States, and South Africa will have alcohol-related disease, and there are indications that this type of disease is increasing [1]. Pain is the cardinal symptom in chronic pancreatitis, and together with the usual problem of contemporary alcoholism, is the most difficult feature to treat [2]. For some patients with these diseases the pain is so severe that all walking hours are devoted to its control; quality of life is very poor [3]. As we are dealing with a devastating disease with unclear basic pathophysiology all contributions to the knowledge of etiology are welcome, especially if new knowledge can be used therapeutically.

It is possibly correct to state that most patients with chronic pancreatitis have a deficiency of selenium. However, the patients with chronic pancreatitis are also smokers that eat little fruit and vegetables, and consume large quantities of alcohol. This can not be disregarded as alcohol per se is correlated to smoking [4, 5]. Also the correlation between an unhealthy diet and alcoholism is strong. These types of confounding factors must be taken into account when discussing potential causal relationships between the dietary influence on pathophysiology.

In alcoholic chronic pancreatitis, cessation of alcohol abuse has an impact on the natural course of the disease. Although impairment of pancreatic function progresses after cessetion in most patients, the progress is slower and less severe [6, 7]. In about 50 percent of patients, cessation of alcohol abuse is accompanied by a decreased severity of pain [7–9]. Patients with early onset idiopathic chronic pancreatitis usually live longer through a long period of severe pain but slowly develop morphological and functional pancreatic damage, whereas patients with late onset idiopathic chronic pancreatitis have a mild and painless course [10, 11]. This must be explained-or at least discussed-in the light of the selenium state presented by Bowrey *et al.*

The mechanisms underlying the generation and perpetuation of the pain in chronic pancreatitis is unsufficiently known. Bockman *et al.* [12] have shown an increase both in the number and diameter of pancreatic nerve fibers in tissues from patients with chronic pancreatitis compared with the normal pancreas and Büchler *et al.* [13] found that the pattern of intrinsic and possibly extrinsic innervation of the pancreas is changed in chronic pancreatitis. This leads to a differential expression of neuropeptides such as substance P and vasoactive intestinal peptide in the chronically inflamed pancreas. Moreover, Nelson *et al.* [14] have reported that changes in levels of methionine-enkephalin correlates with pain, and recently it was found that immune cell infiltration and growth-associated protein 43 expression correlate with pain in chronic pancreatitis [15]. In the future, these facts must be taken into account not only when tayloring the treatment for the patient with chronic pancreatitis but also when discussing etiology. So far the connexions between the dietary selenium deficiency and the nerve injuries have not been well elucidated, which means that even though the concept of oxidative stress seems well founded, much more remains to be done in this research field.

Åke Andrén-Sandberg
Department of Surgery,
Haukeland University Hospital,
Bergen, Norway

### References

[1] Mössner, J. (1993). Epidemiology of Chronic Pancreatitis. In: Beger, H. G., Büchler, M., Malfertheiner, P. (Eds.). Standards in Pancreatic Surgery. Berlin: Springer-Verlag, pp. 263–271.
[2] Leahy, A. L. and Carter, D. C. (1991). Pain and Chronic Pancreatitis. *European Journal of Gastroenterology and Hepatology*, **3**, 425–433.
[3] Andrén-Sandberg, Å. (1997). Pain Relief in Pancreatic Disease. *British Journal of Surgery*, **84**, 1041–1042.
[4] Bourliere, M., Barthet, M., Berthezene, P., Durbec, J. P. and Sarles, H. (1991). Is Tobacco a Risk Factor for Chronic Pancreatitis and Alcohol Cirrhosis? *Gut*, **32**, 1392–1395.
[5] Yen, S., Hsieh, C. C. and MacMahon, B. (1982). Consumption of Alcohol and Tobacco and other Risk Factors for Pancreatitis. *American Journal of Epidemiology*, **116**, 407–412.
[6] Amman, R. W., Akovbiantz, A., Largiader, F. and Schueler, G. (1984). Course and Outcome of Chronic Pancreatitis. Longitudinal Study of a mixed Medical-surgical Series of 245 patients. *Gastroenterology*, **86**, 820–828.
[7] Gullo, L., Barbara, L. and Labo, G. (1988). Effect of Cessation of Alcohol use on the Course of Pancreatic Dysfunction in Alcoholic Pancreatitis. *Gastroenterology*, **95**, 1063–1068.
[8] Little, L. M. (1987). Alcohol Abuse and Chronic Pancreatitis. *Surgery*, **101**, 357–360.
[9] Hayakawa, T., Kondo, T., Shibata, T., Sugimoto, T. and Kitagawa, U. (1989). Chronic Alcoholism and Evolution of Pain and Prognosis in Chronic Pancreatitis. *Digestive Diseases and Science*, **34**, 33–38.
[10] Amman, R. W., Buehler, H., Muench, R., Freiburghaus, A. and Siegenthaler, W. (1987). Difference in the Natural History of Idiapathic (Nonalcoholic) and Alcoholic Chronic Pancreatitis. A comparative Study of 287 patients. *Pancreas*, **2**, 368–377.
[11] Layer, P., Yamamoto, H., Kalthoff, L., Chain, J. E., Bakken, J. and DiMagno, E. P. (1994). The different Courses of Earthly- and Late-onset idiopathic and Alcoholic Chronic Pancreatitis. *Gastroenterology*, **107**, 1481–1487.
[12] Bockman, D. E., Büchler, M., Malfertheiner, P. and Beger, H. (1988). Analysis of Nerves in Chronic Pancreatitis. *Gastroenterology*, **94**, 1459–1469.
[13] Büchler, M., Weihe, E., Friess, H., Malfertheiner, P., Bockman, D. E., Müller, S., Nohr, D. and Beger, H. G. (1992). Changes in Peptidergic Innervation in Chronic Pancreatitis. *Pancreas*, **7**, 183–192.
[14] Nelson, D. K., Gress, T. M., Lucas, D. L., Müller-Pillasch, F., Di Sebastino, P., Friess, H., Adler, G., Büchler, M. and Malfertheiner, P. (1993). Gene Expression and Posttranslational Processing of Intrapancreatic Proenkephalin: a Link to Pancreatic Pain in Chronic Pancreatitis? (abstr.) *Gastroenterology*, **104**, A323.
[15] Di Sebastiano, P., Fink, T., Weihe, E., Friess, H., Innocenti, P., Beger, H. G. and Büchler, M. W. (1997). Immune Cell Infiltration and Growth-associated Protein 43 Expression Correlate with Pain in Chronic Pancreatitis. *Gastroenterology*, **112**, 1648–1655.

